# Rapid Reductions in Breast Density following Tamoxifen Therapy as Evaluated by Whole-Breast Ultrasound Tomography

**DOI:** 10.3390/jcm11030792

**Published:** 2022-02-01

**Authors:** Gretchen L. Gierach, Mark Sak, Shaoqi Fan, Ruth M. Pfeiffer, Maya Palakal, Cody Ramin, Lisa Bey-Knight, Michael S. Simon, David Gorski, Haythem Ali, Peter Littrup, Mark E. Sherman, Nebojsa Duric

**Affiliations:** 1 Division of Cancer Epidemiology and Genetics, National Cancer Institute, Bethesda, MD 20892, USA; shaoqi.fan@nih.gov (S.F.); pfeiffer@mail.nih.gov (R.M.P.); mmpalakal@gmail.com (M.P.); cody.ramin@nih.gov (C.R.); 2 Delphinus Medical Technologies, 45525 Grand River Avenue, Novi, MI 48374, USA; msak@delphinusmt.com (M.S.); plittrup@delphinusmt.com (P.L.); Nebojsa_Duric@URMC.Rochester.edu (N.D.); 3 Barbara Ann Karmanos Cancer Institute, 4100 John R, Detroit, MI 48201, USA; beyl@karmanos.org (L.B.-K.); simonm@karmanos.org (M.S.S.); gorskid@med.wayne.edu (D.G.); 4 School of Medicine, Wayne State University, Detroit, MI 48201, USA; 5 Henry Ford Cancer Institute, Henry Ford Health System, 2799 W Grand Boulevard, Detroit, MI 48202, USA; HALI1@hfhs.org; 6 Diagnostic Radiology, Ascension Providence Rochester Hospital, Rochester, MI 48307, USA; 7 Quantitative Health Sciences, Mayo Clinic, 4500 San Pablo Road, Jacksonville, FL 32224, USA; Sherman.Mark@mayo.edu; 8 Department of Imaging Sciences, University of Rochester, Rochester, NY 14642, USA

**Keywords:** breast density, breast neoplasms, tamoxifen, ultrasonography, tomography, chemoprevention

## Abstract

Purpose: Women whose mammographic breast density declines within 12–18 months of initiating tamoxifen for chemoprevention or adjuvant treatment show improved therapeutic responses compared with those whose density is unchanged. We tested whether measuring changes in sound speed (a surrogate of breast density) using ultrasound tomography (UST) could enable rapid identification of favorable responses to tamoxifen. Methods: We evaluated serial density measures at baseline and at 1 to 3, 4 to 6, and 12+ months among 74 women (aged 30–70 years) following initiation of tamoxifen for clinical indications, including an elevated risk of breast cancer (20%) and diagnoses of in situ (39%) or invasive (40%) breast carcinoma, enrolled at Karmanos Cancer Institute and Henry Ford Health System (Detroit, MI, USA). For comparison, we evaluated an untreated group with screen negative mammography and frequency-matched on age, race, and menopausal status (*n* = 150), at baseline and 12 months. Paired *t*-tests were used to assess differences in UST sound speed over time and between tamoxifen-treated and untreated patients. Results: Sound speed declined steadily over the 12 month period among patients receiving tamoxifen (mean (SD): −3.0 (8.2) m/s; *p* = 0.001), whereas density remained unchanged in the untreated group (mean (SD): 0.4 (7.1) m/s; *p* = 0.75 (relative change between groups: *p* = 0.0009)). In the tamoxifen group, we observed significant sound speed reductions as early as 4–6 months after tamoxifen initiation (mean (SD): −2.1 (6.8) m/s; *p* = 0.008). Sound speed reductions were greatest among premenopausal patients (P-interaction = 0.0002) and those in the middle and upper tertiles of baseline sound speed (P-interaction = 0.002). Conclusions: UST can image rapid declines in sound speed following initiation of tamoxifen. Given that sound speed and mammographic density are correlated, we propose that UST breast imaging may capture early responses to tamoxifen, which in turn may have utility in predicting therapeutic efficacy.

## 1. Introduction

Tamoxifen is a selective estrogen receptor modulator which reduces risk of estrogen receptor (ER)-positive breast cancer risk by 50% in the prevention setting for women at high breast cancer risk (e.g., based on the Gail model) [1] and lowers mortality by 30% in the adjuvant setting irrespective of grade [2]. Elevated mammographic breast density is one of the strongest risk factors for breast cancer in the general population [3], and studies suggest that declines in density with tamoxifen use may represent a marker of efficacy in both prevention and treatment [4,5,6,7]. However, imprecision in measurement of mammographic density renders detection of small changes in density difficult, and serial assessment is limited by repeated radiation exposure. Furthermore, limitations of mammography include the need for breast compression, which distorts architecture and varies between examinations, and which may limit the reproducibility of two-dimensional measurements of breast density.

To assess breast density repeatedly over time while avoiding ionizing radiation, we used a novel ultrasound tomography (UST) scanner to measure volumetric breast density without compression in women before and during their first year of tamoxifen use for clinical indications, including chemoprevention and treatment. UST examination calculates sound speed within the breast, an objective physical measurement that is directly related to tissue density [8,9,10,11,12]. As units of sound speed are fixed to an external standard, UST breast sound speed measurements are relatively unaffected by day-to-day performance factors [13]. UST whole-breast sound speed estimates have been previously shown to be highly reproducible surrogate measures of volumetric breast density [14,15,16,17,18,19], which are strongly and positively associated with breast cancer risk [17]. The specific goal of this project was to utilize UST to describe the early time course of tamoxifen-associated volumetric breast density declines. The broader objective was to assess the concept of breast density decline as a biosensor of tamoxifen response and UST as a useful tool for making this determination. For comparison, we assessed a group of matched untreated women with screen negative mammography at a 12 month interval.

## 2. Materials and Methods

### 2.1. Study Population

The Ultrasound Study of Tamoxifen enrolled 82 patients referred for tamoxifen therapy for clinical indications and a matched comparison group of 165 untreated women with screen negative mammograms and no personal history of breast cancer, aged 30–70 years, at the Barbara Ann Karmanos Cancer Institute (KCI) and Henry Ford Health System (HFHS) in Detroit, MI, USA from 2011 to 2014 [13,17]. Patients were ineligible for study enrollment if they were pregnant, were lactating, had active skin infections or open chest wounds, had a breast size more than 22 cm in diameter (limit of the size of the ring ultrasound transducer), or weighed over 350 lb (weight limit, as specified by the manufacturer of the UST table). Furthermore, ineligible patients included those with breast implants or reduction mammoplasty or who were taking endogenous hormones at the time of study enrollment (i.e., oral contraceptives and menopausal hormone therapy), because the use and discontinuation of these medications may alter breast density [20].

For the tamoxifen-treated group, patients were identified at KCI and HFHS prior to tamoxifen initiation from a health professional’s referral based on their elevated risk of breast cancer, including a diagnosis of atypical lobular or atypical ductal hyperplasia (ALH/ADH), ductal carcinoma in situ (DCIS), lobular carcinoma in situ (LCIS), or unilateral invasive breast cancer. Women with a prior cancer diagnosis in the breast contralateral to their diagnosis at the time of study referral were ineligible; similarly, women with a diagnosis of bilateral synchronous breast cancer were ineligible for the tamoxifen group, as a breast without cancer would not have been available for analysis. In order to assess whether any observed changes in UST density over time were greater than the minimal declines in breast density that might be observed with aging in the absence of tamoxifen exposure, we identified an untreated group from patients undergoing screening mammography at KCI or HFHS with the recommendation to continue routine screening (i.e., Breast Imaging Reporting and Data System (or BI-RADS) diagnostic score of “1” or “2”). In addition, these women had not received medication or radiation for cancer and were not taking tamoxifen or raloxifene to lower breast cancer risk. Untreated women were invited to undergo UST and were frequency-matched to the case group on the basis of age (within 1 year categories where possible), race, and menopausal status. A previous analysis in this study population compared baseline measures of breast sound speed and mammographic breast density in tamoxifen-treated and untreated patients, with findings demonstrating that baseline measures of UST sound speed were more strongly associated with breast cancer risk than mammographic breast density [17]. The focus of the present analysis was to assess differences in UST sound speed over time and between tamoxifen-treated and untreated patients.

For the purposes of this analysis, *n* = 6 women in the group recommended to receive tamoxifen who did not initiate therapy were excluded. In addition, the analysis was restricted to the ~90% of participants who completed the baseline UST scan and at least one follow-up UST scan, resulting in a final analytic population of *n* = 74 in the tamoxifen group and *n* = 150 in the untreated group (Figure S1). Informed consent was obtained from all participants, and the study protocol was approved by the Institutional Review Boards at KCI, HFHS, and the NCI.

### 2.2. Breast Sound Speed Assessment

**Figure 1:**
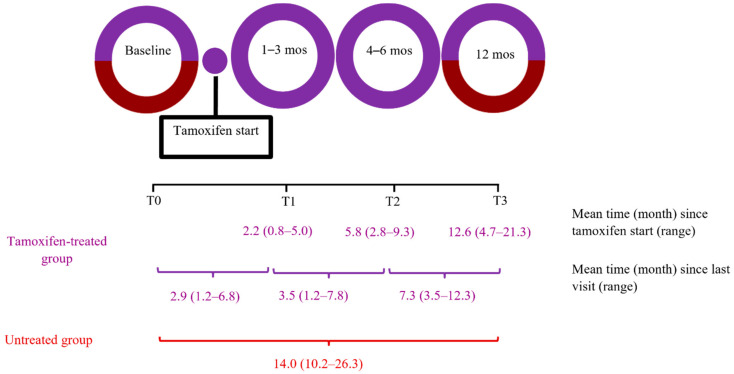
Assessing interval changes in volumetric breast density with ultrasound tomography in the Ultrasound Study of Tamoxifen. Purple = tamoxifen-treated group (*N* = 74); maroon = untreated group (*N* = 150).

Among the tamoxifen-treated patients, a baseline UST scan was completed prior to tamoxifen initiation (T0) and at approximately 1–3 months (T1), 4–6 months (T2), and 12 months (T3) post tamoxifen initiation (Figure 1). Among those breast cancer patients diagnosed with high-risk breast lesions or cancer, UST was completed for the contralateral breast to avoid the potential influences of biopsy changes on breast sound speed. For breast cancer patients receiving neoadjuvant chemotherapy prior to initiating tamoxifen therapy (*n* = 7), the sequence of the four UST scans was modified in an attempt to isolate the influence of tamoxifen on breast UST sound speed as follows: prior to treatment, post chemotherapy and prior to tamoxifen (defined as T0 for the purposes of the present analysis), and at approximately 1–3 months (T1) and 12 months (T3) post tamoxifen initiation. The untreated group underwent two UST scans, at baseline and at a 12 month follow-up visit. For this group, we randomly selected a breast for UST assessment and imaged the same breast at baseline and follow-up, since concurrent mammographic density measurements of left and right breasts from the same individuals have been reported to be highly correlated [21].

UST scans were performed at KCI with the SoftVue system, a clinical prototype manufactured by Delphinus Medical Technologies (Novi, MI, USA), cleared by the FDA for clinical use, and recently approved for breast cancer screening [13,17,19]. Patients were scanned in the prone position with the breast immersed in a water bath. UST uses a 22 cm ring-shaped ultrasound transducer, consisting of 2048 elements that transmit and receive ultrasound pulses. The transducer is mounted on an automated gantry that progressively captures 40–100 coronal image slices beginning at the chest wall and progressing to the nipple, requiring about 90 s to capture the raw data. Images were reconstructed from the raw data and output in DICOM format. The volume averaged sound speed (VASS, m/s) of the breast, a surrogate of volumetric breast density [14,15,16,17,18,19], was calculated using techniques described previously [8,13,17]. Briefly, to minimize background noise, a segmentation algorithm was utilized to remove sections of each image associated with the water bath. To analyze change in sound speed between serial scans, image files were restricted to a common volume that was contained within all scans [13]. For tomograms included in the common volume, the sound speed measures, in m/s, were calculated through a direct count of pixel values in each tomogram using automated scripts and were averaged together for the image stack to generate the VASS measurement used as a proxy of breast density. Observers were blinded to tamoxifen status for UST sound speed measurements. A previous reproducibility study demonstrated that UST VASS estimates were highly reliable for a single timepoint (intraclass correlation coefficient, ICC = 93.4%) and had very good reproducibility for changes in VASS (ICC = 70.4%) [13].

### 2.3. Covariates

At the time of each UST scan, a standard health history questionnaire was administered by a research nurse to collect demographics, reproductive history, menopausal status, and medication use. Measured height was collected at baseline and measured weight was collected at each UST scan to compute body mass index (BMI, kg/m^2^) and to evaluate change in BMI over time.

### 2.4. Statistical Analysis

Participant characteristics between the tamoxifen-treated and untreated groups were compared using chi-square or Fisher’s exact tests for categorical variables and the Wilcoxon rank sum test for continuous variables. Mean change in VASS from baseline to follow-up was approximately normally distributed in both groups. We evaluated mean change in VASS by participant characteristics using paired *t*-tests or Kruskal–Wallis tests as appropriate. In sensitivity analyses, we adjusted for weight change at 12 months (tertiles) using generalized linear regression models. Changes in VASS measures over time were evaluated separately among cases and controls using paired *t*-tests. To compare the average change in VASS over the 12 month follow-up period between cases and controls, a two-sample *t*-test was used.

Relative change in VASS in the tamoxifen-treated versus untreated groups was assessed with generalized linear regression models that accounted for repeated measures from the same woman in the variance calculation [22]. The models included time since tamoxifen initiation (in months) and the matching factors; we additionally tested for effect modification of this relationship by menopausal status and baseline VASS. Sensitivity analyses were conducted excluding breast cancer patients who underwent neoadjuvant chemotherapy, who became postmenopausal by 12 month visit, or who self-reported tamoxifen cessation at any visit over study follow-up. Analyses were performed using SAS V9.4 (SAS, Cary, NC, USA). We compared regression coefficients for time from the separate GLM models using chi-squared tests to obtain *p*-values for interaction (test statistics were calculated in R). Statistical tests were two-sided, and *p*-values < 0.05 were considered statistically significant.

## 3. Results

Of the 74 tamoxifen-treated patients, 96% completed the T1 scan (1–3 months post tamoxifen initiation), 89% completed the T2 scan (4–6 months post tamoxifen initiation) and 95% completed the T3 scan (12 months post tamoxifen initiation). Indications for tamoxifen were as follows: 15 (20%) were high-risk patients (i.e., without histologically confirmed in situ or invasive breast cancer), 29 (39%) were diagnosed with in situ carcinoma, and 30 (40%) were diagnosed with invasive breast cancer, including *n* = 7 who were prescribed neoadjuvant chemotherapy. Furthermore, *n* = 2 tamoxifen-treated patients (3%) reported stopping tamoxifen prior to T2, and *n* = 4 tamoxifen-treated patients (6%) reported stopping tamoxifen prior to T3.

Participant characteristics are shown in Table 1. No statistically significant differences were observed between groups in matching factors (i.e., age, race and menopausal status). Median (range) age at baseline was 51 (30–70) years among tamoxifen-treated patients and 51 (33–69) among untreated patients. Slightly over 50% of women in both groups were Black and premenopausal and over 75% were overweight or obese. Weight change was minimal over the study period in both groups. Compared with untreated women, patients receiving tamoxifen were more likely to have a first degree relative with breast cancer (32% vs. 20%; *p* = 0.04) and to have elevated baseline VASS (*p* = 0.01), as previously reported in this study population [17].

The median (range) time between the baseline UST scan (T0) and the follow-up UST scan (T3) was 13 (7–21) months in the treated and 13 (10–26) months in the untreated groups. Associations between participant characteristics and change in VASS from baseline (T0) to the T3 follow-up scan are presented in Table 2. In both groups, weight gain at T3 was associated with a statistically significant reduction in VASS at T3 (*p* ≤ 0.02). Among tamoxifen-treated women, premenopausal status at baseline was associated with a statistically significant reduction in VASS at T3 (mean (SD): −4.2 (9.0) m/s; *p* = 0.02). On average, greater decreases in VASS from baseline to T3 were observed for patients with higher baseline VASS in both treated (*p* = 0.0001) and untreated women (*p* = 0.04). Observed risk factor relationships were attenuated slightly with adjustment for weight change between T0 and T3 (Table 2).

**Figure 2:**
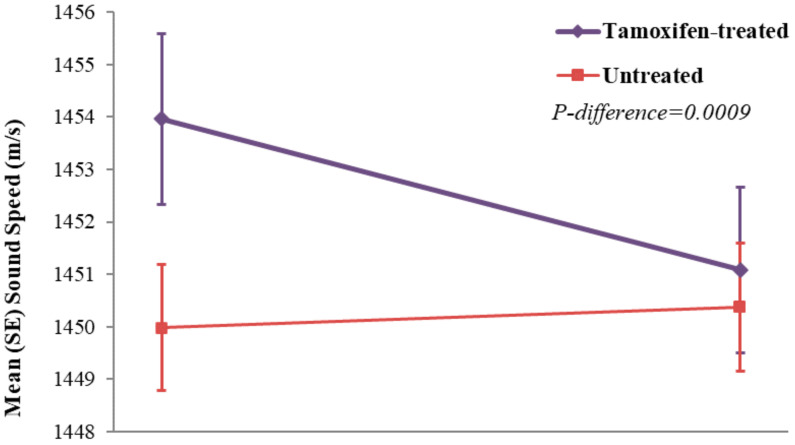
Mean (SE) reductions in breast volume average sound speed between baseline and 12 months by tamoxifen-treated (*N* = 74) and untreated (*N* = 150) study groups, in the Ultrasound Study of Tamoxifen.

At the T3 scan, VASS was statistically significantly reduced in the tamoxifen-treated group (mean (SD): −3.0 (8.2) m/s; *p* = 0.0015), while VASS remained stable in the untreated group (mean (SD): 0.4 (7.1) m/s; *p* = 0.75) (Figure 2). The difference in density change between groups was statistically significant (*p* = 0.0009).

**Figure 3:**
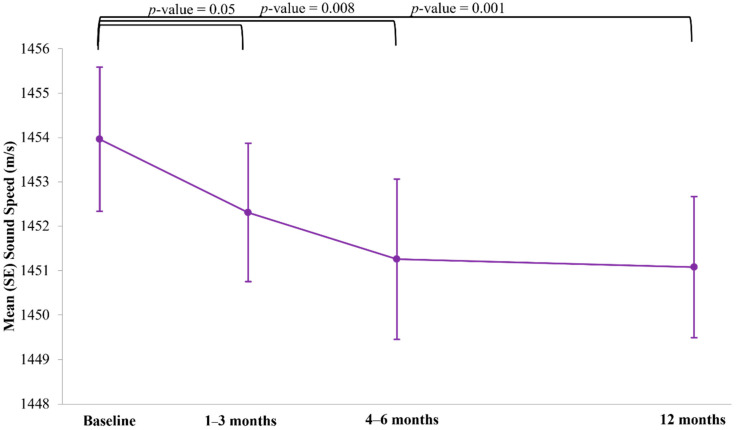
Mean (SE) reductions in breast volume average sound speed by study visit in the tamoxifen-treated group (*N* = 74), in the Ultrasound Study of Tamoxifen.

**Figure 4:**
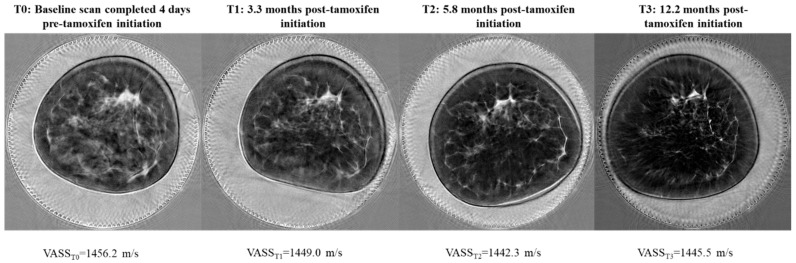
Example of single ultrasound tomography sound speed images from the breast contralateral to a diagnosis of invasive breast cancer in a tamoxifen-treated premenopausal patient over the course of the study follow-up period. Dense tissue appears white on the image with higher sound speed values, while fatty tissue appears dark with lower sound speed values. Among the tamoxifen-treated group, we observed significant VASS declines as early as 4–6 months post tamoxifen initiation (T2), with the strongest results observed among premenopausal patients.

In analyses restricted to the tamoxifen-treated group, we observed significant reductions in VASS as early as 4–6 months after initiation of drug (Figure 3, mean (SD): −2.1 (6.8) m/s; *p* = 0.008). Results from linear regression models adjusted for matching factors indicated that, for every month post tamoxifen initiation, VASS decreased by −0.31 m/s (95% confidence interval (CI): −0.47, −0.14; *p* = 0.0004). Example UST sound speed images from a tamoxifen-treated premenopausal patient are shown in Figure 4. VASS reductions were greatest among premenopausal tamoxifen-treated women (Figure S2, P-interaction = 0.0002) and those in the middle and upper tertiles of baseline VASS (P-interaction = 0.002). Results from sensitivity analyses excluding seven tamoxifen-treated patients who underwent neoadjuvant chemotherapy, four premenopausal tamoxifen-treated patients who underwent menopause by the end of study follow-up, and four tamoxifen-treated patients who self-reported cessation of tamoxifen over the study follow-up period were consistent with the overall study results.

## 4. Discussion

Using novel nonionizing whole-breast UST to repeatedly assess VASS, a reliable and accurate surrogate of volumetric breast density, we found that VASS decreased steadily and rapidly within the first year of tamoxifen therapy, while remaining stable in an untreated matched comparison group. Furthermore, we observed significant VASS declines as early as 4–6 months post tamoxifen initiation, with strongest results among premenopausal patients. These findings suggest that UST may have utility in monitoring early changes in VASS as a potential biomarker of clinical tamoxifen response. Further studies are needed to assess underlying mechanisms and to determine whether this observation can predict clinical outcome, as has been shown for decline in mammographic density.

Our UST findings are consistent with prior studies demonstrating that tamoxifen can induce decreases in mammographic breast density in both high-risk women and breast cancer patients [4]. Previous studies have shown that women whose mammographic breast density declines 12–18 months after receiving tamoxifen for chemoprevention or adjuvant treatment are more likely to gain benefit than those whose breast density does not decline [5,6,7]. In the IBIS-1 chemoprevention trial of tamoxifen vs. placebo, women in the tamoxifen arm experienced a significantly greater decline in density as compared with placebo at 5 years (13.7% vs. 7.3%, respectively; *p* < 0.001). About two-thirds of the reduction in density attained with tamoxifen occurred within 18 months (7.9% vs. 3.5%, *p* < 0.001) [23]. Subsequent data from IBIS-1 indicated that successful chemoprevention with tamoxifen use was strongly related to a decline in mammographic density by ~10% or more at 12–18 months [24]. Mammographic density decline 12–18 months post tamoxifen initiation has also been linked to reductions in breast cancer recurrence and improved breast cancer-specific survival [5,6,7].

We observed a statistically significant reduction in VASS as early as 4–6 months post tamoxifen initiation. Given that screening was performed annually in IBIS-1, the trial was unable to assess whether density declines associated with tamoxifen use occurred earlier than 1 year, and most prior studies evaluated density change at yearly intervals [5,6,7]. Consistent with our findings, however, a recent report from a 6 month low-dose tamoxifen trial from the Swedish KARMA Cohort identified significant reductions in mammographic breast density with both 2.5 and 20 mg doses of tamoxifen, primarily in premenopausal women [25]. Future work is needed to determine whether low-dose tamoxifen, which confers fewer side-effects than the standard 20 mg dose [26], in concert with early tamoxifen-associated declines in breast density, may predict therapeutic effectiveness. UST may be a valuable low-risk and reliable tool for rapidly determining which patients are responding to therapy, which would have important clinical implications as tamoxifen uptake and adherence remain low due to concerns about toxicities and vasomotor symptoms [27]. Furthermore, defining a biomarker of effectiveness could potentially suggest a path to precision dosing and reducing side-effects.

It is unclear why tamoxifen-associated density declines appear to be greater in premenopausal as compared with postmenopausal women, despite the beneficial chemopreventive and adjuvant effects of tamoxifen therapy irrespective of menopausal status [2,28]. As postmenopausal women tended to have lower baseline breast density as compared with premenopausal women, there was a smaller range of densities available post menopause in which to measure declines. It has been hypothesized that breast density decline represents a biosensor of tamoxifen response, reflecting estrogen suppression and the bioavailability and action of active drug metabolites [5,29]. Future studies of serial circulating and tissue-based markers collected pre and post tamoxifen may provide mechanistic insights into the biologic underpinnings of tamoxifen-associated density declines and related reductions in breast cancer risk and progression.

Strengths of this study include the longitudinal study design with the use of a novel 3D UST imaging device to repeatedly assess VASS, a previously defined surrogate of breast density [14,15,16,17,18], in well-characterized tamoxifen-treated and untreated matched comparison groups. UST is well suited for this application because it produces volumetric data and avoids artefacts secondary to breast compression and exposure to potentially harmful ionizing radiation. As VASS is an objective physical measurement that is fixed to an external standard, UST VASS is relatively unaffected by day-to-day performance factors and has demonstrated reliability for measuring density change [13]. Furthermore, in this study population, risk factors associated with density declines (e.g., premenopausal status, elevated baseline density) were consistent with those reported in prior studies using mammography to assess density change [23,25,30], lending validity to our findings. Although limited in sample size, within-person comparisons reduced the potential for confounding. Lastly, this was a short-term longitudinal study with change in VASS as a biomarker endpoint. Prior studies have observed that mammographic breast density measurements approximately 1 year after tamoxifen initiation are generally representative of longer-term changes [31,32]. Future work is needed to evaluate the persistence of effects of UST sound speed declines on breast cancer outcomes to establish VASS as a clinically relevant biomarker.

In summary, we observed that UST measurement of VASS enables detection of rapid declines after tamoxifen initiation, suggesting the potential to use VASS as an early marker of tamoxifen response. Further studies are needed to assess whether patterns of change in VASS over time among tamoxifen-treated patients can discriminate preventive or adjuvant benefit.

## Figures and Tables

**Table 1:** Characteristics of tamoxifen-treated and untreated patients without a personal history of breast cancer, in the Ultrasound Study of Tamoxifen.

	Tamoxifen-Treated Group (*N* =74)		Untreated Group (*N* = 150)		*p*-Value *
Characteristic	*N*	%	*N*	%	
Age at baseline—Median (Range)	51.3	(30, 71)	51.1	(33, 69)	0.68 **
Age at baseline					
<45	17	23.0	32	21.3	0.97
45–50	17	23.0	36	24.0	
50–55	17	23.0	32	21.3	
≥55	23	31.1	50	33.3	
Race					
White	26	35.1	46	30.7	0.10
Black	41	55.4	99	66.0	
Other	7	9.5	5	3.3	
Hispanic					
No	70	94.6	146	97.3	0.44 ^†^
Yes	4	5.4	4	2.7	
BMI at baseline					
<25	16	21.6	32	21.3	0.76
25–30	24	32.4	42	28.0	
30+	34	45.9	76	50.7	
BMI at 12 month follow-up					
<25	17	24.3	33	22.0	0.31
25−30	24	34.3	39	26.0	
30+	29	41.4	78	52.0	
Weight change (lbs)—median (IQR)	1	(−4.2, 5.7)	−0.1	(−4.6, 4.8)	0.51
Weight change at 12 months (lbs)					
<−2.4	26	37.1	51	34.0	0.15
−2.4–3.4	15	21.4	51	34.0	
≥3.4	29	41.4	48	32.0	
Education					
At most, high school/GED	23	31.1	43	28.7	0.93
Some college/postsecondary courses	24	32.4	51	34.0	
College/graduate school	27	36.5	56	37.3	
Age at menarche					
≤12	41	55.4	86	57.7	0.44
13	14	18.9	35	23.5	
14+	19	25.7	28	18.8	
Age at first birth					
Nulliparous	11	14.9	35	23.3	0.10
<18	7	9.5	25	16.7	
18–21	13	17.6	27	18.0	
22–27	17	23.0	32	21.3	
27+	26	35.1	31	20.7	
Menopausal status					
Premenopausal	49	66.2	84	56.0	0.14
Postmenopausal	25	33.8	66	44.0	
Any first-degree relative with breast cancer					
No	50	67.6	120	80.0	**0.04**
Yes	24	32.4	30	20.0	
Quartiles of baseline sound speed (m/s)					
<1440.63	7	9.5	39	26.0	**0.01**
1440.63–1445.65	17	23.0	39	26.0	
1445.65–1452.81	20	27.0	35	23.3	
≥1452.81	30	40.5	37	24.7	

*p*-values < 0.05 are in bold font. * *p*-Values from chi-square test except where noted; ** *p*-values from Wilcoxon rank sum test; ^†^ *p*-values from Fisher’s exact test. BMI, body mass index; IQR, interquartile range; MHT, menopausal hormone therapy.

**Table 2:** Mean change in sound speed (m/s) from baseline to 12 month follow-up in the tamoxifen-treated and untreated groups by participant characteristics, in the Ultrasound Study of Tamoxifen.

	Tamoxifen-Treated Group (*N* = 74)			Untreated Group (*N* = 150)		
Characteristic	Mean (SD)	*p*-Value *	*p*-Value ^†^	Mean (SD)	*p*-Value *	*p*-Value ^†^
Age at baseline						
<45	−1.9 (10.9)	0.51	0.58	0.5 (6.2)	0.66	0.64
45–50	−4.7 (7.6)			0.8 (7.9)		
50–55	−3.9 (8.4)			−0.6 (9.0)		
≥55	−1.8 (6.2)			0.7 (5.7)		
Race						
White	−4.0 (8.9)	0.99	0.73	1.0 (5.7)	0.83	0.38
Black	−2.5 (8.0)			0.0 (7.7)		
Other	−2.4 (7.3)			2.1 (4.6)		
Hispanic						
No	−3.2 (8.2)	0.79 **	0.28	0.4 (7.2)	0.62 **	0.41
Yes	0.7 (7.6)			1.5 (2.7)		
BMI at baseline						
<25	−6.7 (9.8)	0.42	0.31	−0.1 (8.8)	0.40	0.34
25–30	−2.1 (6.7)			−0.6 (8.9)		
30+	−2.1 (8.2)			1.1 (4.8)		
BMI at 12 month follow-up						
<25	−4.4 (10.0)	0.89	0.50	0.7 (9.3)	0.89	0.68
25–30	−3.1 (6.3)			0.3 (8.7)		
30+	−2.1 (8.5)			0.3 (4.9)		
Weight change at 12 months (lbs)						
<−2.4	0.2 (7.9)	**0.02**	–	2.0 (8.3)	**0.0004**	–
−2.4–3.4	−2.5 (8.1)			1.2 (5.7)		
≥3.4	−6.2 (7.5)			−2.2 (6.5)		
Education						
At most, high school/GED	−2.4 (7.2)	0.79	0.77	−0.4 (8.9)	0.53	0.46
Some college/postsecondary courses	−2.0 (9.1)			−0.0 (7.0)		
College/graduate school	−4.3 (8.4)			1.4 (5.4)		
Age at menarche						
≤12	−2.9 (9.1)	0.25	0.32	0.6 (7.3)	0.63	0.49
13	−0.5 (5.2)			1.1 (5.4)		
14+	−5.0 (7.5)			−1.1 (8.2)		
Age at first birth						
Nulliparous	−4.1 (8.1)	0.40	0.64	0.4 (8.7)	0.89	0.8
<18	−2.4 (2.9)			0.4 (9.5)		
18–21	0.3 (7.3)			1.0 (4.6)		
22–27	−3.4 (5.9)			−0.5 (5.8)		
27+	−4.1 (10.5)			0.8 (6.0)		
Menopausal status						
Premenopausal	−4.2 (9.0)	0.02 **	0.09	0.5 (6.7)	0.60 **	0.48
Postmenopausal	−0.6 (5.5)			0.2 (7.5)		
Any first degree relative with breast cancer						
No	−3.0 (8.3)	0.49 **	0.81	0.2 (7.0)	0.23 **	0.40
Yes	−3.0 (8.1)			1.3 (7.6)		
Quartiles of baseline sound speed (m/s)						
<1440.63	2.1 (3.3)	**0.0001**	**0.013**	2.3 (3.2)	**0.04**	0.06
1440.63–1445.65	0.9 (3.6)			1.7 (5.1)		
1445.65–1452.81	−2.1 (5.2)			−1.1 (6.3)		
≥1452.81	−7.2 (10.4)			−1.5 (11.0)		

* *p*-Values from Kruskal–Wallis test except where noted; *p*-values less than 0.05 are in bold font; ** *p-*values from paired *t*-test; ^†^ *p*-values from generalized linear regression modeling after adjusting for weight change at 12 months (tertiles); *p*-values less than 0.05 are in bold font. BMI, body mass index; MHT, menopausal hormone therapy; SD, standard deviation.

## Data Availability

The data presented in this study are available upon reasonable request from the corresponding author and with permission from KCI and HFHS.

## Supplementary Materials

The following are available online at https://www.mdpi.com/article/10.3390/jcm11030792/s1, Figure S1: Overview of study visits and exclusion criteria, the Ultrasound Study of Tamoxifen, Figure S2: Mean (SE) reductions in breast volume average sound speed by menopausal status in the tamoxifen-treated group (*N* = 74), the Ultrasound Study of Tamoxifen.
