# Supplementary material for: Rapid Reductions in Breast Density following Tamoxifen Therapy as Evaluated by Whole-Breast Ultrasound Tomography
Source: J Clin Med. 2022 Feb 1;11(3):792. doi: 10.3390/jcm11030792 (PMC8836554; doi:10.3390/jcm11030792)
Supplement: Supplementary file 1 [file jcm-11-00792-s001.zip › jcm-1509444-supplementary.pdf]

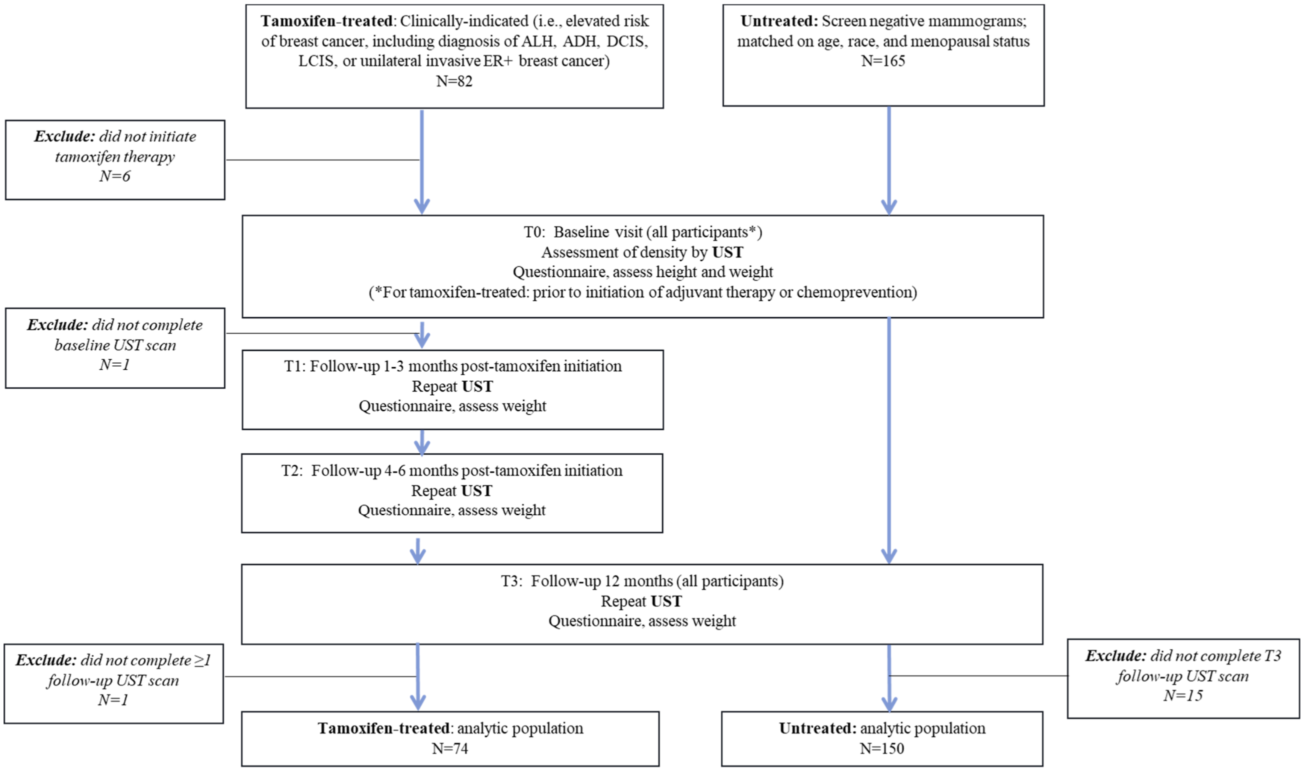

Supplementary Figure S1. Overview of study visits and exclusion criteria, the Ultrasound Study of Tamoxifen.

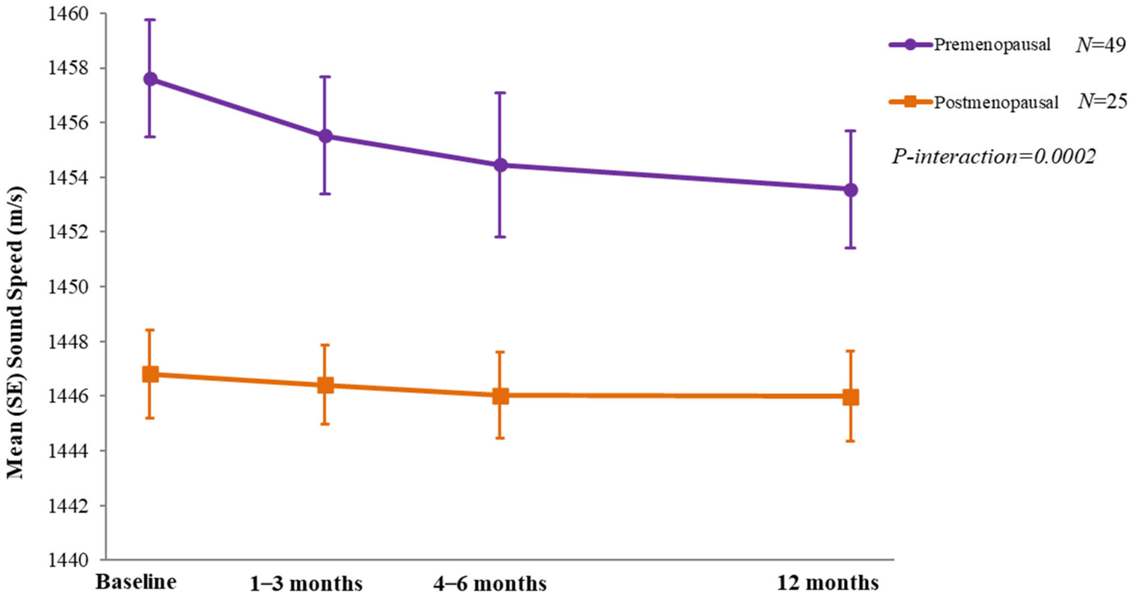

Supplementary Figure S2. Mean (SE) reductions in breast volume average sound speed by menopausal status in the tamoxifen-treated group (N=74), the Ultrasound Study of Tamoxifen
